# Protective Effects of Konjac and Inulin Extracts on Type 1 and Type 2 Diabetes

**DOI:** 10.1155/2019/3872182

**Published:** 2019-10-07

**Authors:** Tianle Gao, Yue Jiao, Yang Liu, Tao Li, Zhiguo Wang, Danqiao Wang

**Affiliations:** ^1^State Key Laboratory of Bioactive Substances and Function of Natural Medicine, Institute of Materia Medica, Chinese Academy of Medical Sciences, Beijing 100050, China; ^2^Beijing Key Laboratory of Traditional Chinese Medicine Basic Research on Prevention and Treatment of Major Diseases, Experimental Research Center, China Academy of Chinese Medical Sciences, Beijing 100000, China

## Abstract

**Objective:**

The present study was designed to determine whether konjac and inulin extracts or their combination, konjac-inulin (KI) composition, as diet supplementary, can exert beneficial effects against type 1 diabetes and type 2 diabetes using animal models.

**Methods:**

A total of 60 diabetic (type 1) rats induced by streptozotocin (STZ) were randomly assigned to five groups: vehicle control (STZ group), KI combination at low dose group (KI-L group), KI combination at medium dose group (KI-M group), KI combination at high dose group (KI-H group), konjac extract group (konjac group), and inulin extract group (inulin group). A sham group (without STZ) was also included. Levels of blood glucose were monitored at each week. After continuous treatment of each diet for 24 days, a glucose tolerance test was performed. After 28 days of treatment, plasma biochemical indicators including glycated serum proteins, total cholesterol, and triglycerides were measured and immunohistochemistry staining of the rat pancreas was performed, to study the insulin expressions. Type 2 diabetes was developed in db/db mice. A total of 28 db/db mice were divided into 4 groups: vehicle control (db/db group), KI composition group (KI group), konjac extract group (konjac group), and inulin extract group (inulin group). A wild-type control group (wild-type group) for db/db mice was also included. Levels of blood glucose, body weight, and blood triglycerides were monitored at each week.

**Results:**

Daily use of the KI composition significantly decreased levels of blood glucose and blood triglycerides, as well as improved the insulin production in islets or reduced development of obesity in STZ-induced diabetic rats or in db/db mice. Such effects from KI composition were better than single ingredient of konjac or inulin extract.

**Conclusion:**

The results of this study suggest that daily use of KI composition has a protective role on type 1 and 2 diabetes and provided experimental basis for further development of KI composition as a food supplement for diabetic or diabetic high-risk population.

## 1. Introduction

Diabetes mellitus (DM) is a global health challenge. In recent decades, the change of lifestyle towards expending patterns of those with obesity, especially unhealthy diet combined with less physical exercise, is a key factor leading to DM [1]. On the contrary, in a large-scale retrospective study, diet high in cereal fiber and low in glycemic load and trans fat was found to bare a protective role against the development of type 2 diabetes [2], indicating the importance of diet in DM prevention and therapy.

*Amorphophallus konjac*, commonly known as konjac, is a perennial herb, which contains about 35% starch, 30% konjac glucomannan (dietary fiber), 5%-10% crude protein, and a variety of vitamins and mineral elements including potassium, phosphorus, and selenium [3]. Through mitigating the nutritional imbalance by increasing dietary fiber intake, konjac is beneficial to various disorders such as constipation, high levels of blood fat/glucose, and overweight [3]. Additionally, konjac extracts were associated with nitric oxide free radical regulatory effect and positively decreased STZ-induced nitric oxide upregulation in islets and improved insulin production [4].

Inulin is a soluble dietary fiber, which is stored in the tubers of a perennial herb *Jerusalem artichoke*. Due to the specific chemical structure, inulin dietary fiber is not digested in the human mouth, stomach, or small intestine, so that it does not cause increase in blood glucose concentration, but rather prevents sharp changes in blood sugar and protecting islet cells after ingestion, thus known as “natural insulin” for diabetic patients [5]. In 2017, a systematic review of clinical trial results showed that dietary supplementation with inulin reduced biomarkers of metabolic syndrome [6]. In the United States in 2018, the Food and Drug Administration approved inulin as a dietary fiber ingredient used to improve the nutritional value of manufactured food products [7].

Since DM is known to be influenced by diet [2] and konjac or inulin dietary fiber may have a protective role against DM, this study is aimed at assessing the efficacy of konjac, inulin extracts, or its combination, konjac-inulin (KI) composition, in STZ-induced rats with type 1 diabetes or db/db mice with type 2 diabetes, by studying the levels of blood glucose and plasma biochemical indicators, the development of obesity, and histopathological alterations of the pancreas.

## 2. Materials and Methods

### 2.1. Materials

In making konjac oral solution, konjac extract (water content < 10%, ash content < 5.0%, and viscosity > 20000 mPa · s, from Shanxi Sciphar Natural Product Co. Ltd., China) with dosage at 1.2 g/kg (in rats) or 1.5 g/kg (in mice) was dissolved in 1.5 mL (in rats) or 0.2 mL (in mice) distilled water, respectively, and applied daily using oral gavage. Inulin extract (water content < 4.5% and ash content < 0.2%, from Shanxi Sciphar Natural Product Co. Ltd., China) was dissolved in drinking distilled water to make 3% inulin drinking solution for replacement of ordinary drinking water in STZ-induced rats. In making konjac and inulin (KI) combinations, konjac extract at low dose of 0.4 g/kg (KI-L), at medium dose of 0.8 g/kg (KI-M), or at high dose of 1.2 g/kg (KI-H) was made into konjac oral solutions using the above-described method and applied daily together with 3% inulin drinking solution (in replacement of the ordinary drinking water), in STZ-induced rats. In db/db mice, konjac extract at 1.5 g/kg (KI) was made into konjac oral solutions and applied daily together with 3% inulin drinking solution.

### 2.2. Animals

The animals used were Sprague-Dawley rats, male, weighing 250-300 g (from Beijing Vital River Laboratory Animal Technology, China), or male BKS.CGg-Dock7^m+/+^Lepr^db/^Nju (db/db) mice with mutation (Lepr^db^)^mut/mut^ and their male wild-type controls (Lepr^db^)^wt/wt^, weighing 20-30 g (from Nanjing Biomedical Research Institute of Nanjing University, China). Animals were housed 4 per cage for rats and 5 per cage for mice at a constant room temperature of 22°C in a 12 : 12 h light-dark cycle with *ad libitum* access to food and water.

### 2.3. Methods

#### 2.3.1. Type 1 Diabetes Model in Rats

After adaptive feeding for 7 days, male SD rats were fasted for 12 hours, blood samples were taken from the tail vein, and the levels of blood glucose were measured using a blood glucometer (Accu-Chek, Roche, Germany). Only rats with a blood glucose level between 5 and 8 mmol/L were selected for induction of type 1 diabetes using streptozotocin (STZ) [8]. In detail, STZ was dissolved in 0.1 M citrate buffer (pH 4.5; 1 mL). Type 1 diabetes was induced by giving a single intraperitoneal (i.p.) injection of STZ at 60 mg/kg. A sham control group received same 0.1 M citrate buffer without STZ. After STZ injection, rats were given 10% (wt/vol) ad libitum fructose solution for 3 days. A blood glucose level was measured after 3 days. Animals with a blood glucose level above 16.7 mmol were considered diabetic and were used in the experiment.

60 STZ-induced rats were randomly divided into six groups (10 rats in each group): group 1 comprised of vehicle control (STZ group), in which STZ-induced diabetic rats were given distilled water (daily, oral gavage) and ordinary drinking water; group 2 included STZ-induced diabetic rats treated with KI combination at low dose (KI-L group), given 0.4 g/kg konjac extract (daily, oral gavage), combined with 3% inulin drinking water; group 3 had STZ-induced diabetic rats treated with KI combination at medium dose (KI-M group), given 0.8 g/kg konjac extract (daily, oral gavage), combined with 3% inulin drinking water; group 4 included STZ-induced diabetic rats treated with KI combination at high dose (KI-H group), given 1.2 g/kg konjac extract (daily, oral gavage), combined with 3% inulin drinking water; group 5 had STZ-induced diabetic rats daily treated with 1.2 g/kg oral konjac extract solution (konjac group), combined with ordinary drinking water; and group 6 included STZ-induced diabetic rats that were given distilled water (daily, oral gavage) combined with 3% inulin drinking water (inulin group). All groups received treatment continuously for 4 weeks. A sham group (10 rats) was treated with distilled water (daily, oral gavage) and ordinary drinking water. The levels of blood glucose were monitored at each week.

#### 2.3.2. Glucose Tolerance Test

A glucose tolerance test was performed in STZ-induced rats with continuous treatment of each diet for 24 days. Animals were fasted for 8 hours and orally administered with 500 mg/kg D-glucose (Merck, USA); blood samples were collected from the tail tip at 0 (baseline), 30, 60, and 120 minutes post D-glucose administration. The levels of blood glucose were measured using a blood glucometer (Accu-Chek, Roche, Germany).

#### 2.3.3. Measurement of Plasma Biochemical Indicators in Rats

After continuous treatment of each diet for 28 days, rats were euthanized with overdose chloral hydrate (10%) and blood samples were collected through abdominal aorta, then centrifuged (3500 r/min) at 4°C for 10 min. Serums were collected and stored at -80°C for the detection of plasma biochemical indicators including glycated serum proteins, total cholesterol, and triglycerides. In detail, the levels of plasma glycated serum proteins were measured using the GSP detection kit (Condor Medical, China), levels of plasma total cholesterol were measured using the Pureauto S CHO-N kit (Sekisui, Japan), and levels of plasma triglycerides were measured using the Pureauto S TG-N kit (Sekisui, Japan). All results were read by a Hitachi 7080 chemistry analyzer (Hitachi, Japan).

#### 2.3.4. Immunohistochemistry Staining of the Rat Pancreas

After continuous diet treatment for 28 days, the pancreas of the rat was taken and the surface blood was washed with physiological saline, fixed in 10% PBS-paraformaldehyde at 4°C for 24 hours. After paraffin embedding and sectioning (5 *μ*m), tissues were stained with hematoxylin-eosin (H-E) and insulin expression in pancreatic tissue was observed with immunohistochemistry. In detail, paraffin-embedded sections were incubated with the primary anti-insulin antibody (1 : 200; host species : mouse; Boster, China) for 24 h at 4°C and subsequently decorated with the secondary antibody (anti-mouse antibody (1 : 200); Boster, China) for 1 h at room temperature (RT); then, reactions were developed using DAB (3,3′-diaminobenzidine) chromogen and DAB substrate buffer (Dako). All images were acquired with a Zeiss Axio Observer epifluorescence microscope.

#### 2.3.5. Type 2 Diabetes Model in Mice

After adaptive feeding for 7 days, male db/db mice (which has long been used for type 2 diabetes research) were fasted for 12 hours, blood samples were taken from the tail vein, and levels of blood glucose were measured using a blood glucometer (Accu-Chek, Roche, Germany). Only mice with a blood glucose level above 10 mmol/L were selected for experiments. 28 db/db mice were divided into 4 groups: group 1 comprised of vehicle control (db/db group), in which db/db mice were given distilled water (daily, oral gavage) and ordinary drinking water; group 2 included db/db mice treated with KI combination (KI group), given 1.5 g/kg konjac extract (daily, oral gavage), combined with 3% inulin drinking water; group 3 had db/db mice daily treated with 1.5 g/kg oral konjac extract solution (konjac group), combined with ordinary drinking water; group 4 included db/db mice that were given distilled water (daily, oral gavage) combined with 3% inulin drinking water (inulin group). All groups received treatment continuously for 6 weeks. The wild-type group (7 mice) was treated with distilled water (daily, oral gavage) and ordinary drinking water. The levels of blood glucose, body weight, and blood triglycerides (using the above-described Pureauto S TG-N kit, Sekisui, Japan) were monitored at each week.

### 2.4. Ethics Statement

All animal studies were performed at the animal facility of Experimental Research Center, China Academy of Chinese Medical Sciences, Beijing, China. Animal experiments were conducted following the National Guidelines for Housing and Care of Laboratory Animals and performed in accordance with the protocol approved by the Research Ethics Committee at China Academy of Chinese Medical Sciences, Beijing, China.

### 2.5. Statistics

Data were presented as mean ± SEM and were analyzed using two-way analysis of variance (ANOVA) with repeated measures or one-way ANOVA, followed by Dunnett's multiple comparisons test or Bonferroni's multiple comparisons test. *P* < 0.05 was considered statistically significant.

## 3. Results

### 3.1. Effect of Konjac, Inulin Extracts, and KI Composition on a Blood Glucose Level of STZ-Induced Diabetic Rats

**Figure 1:**
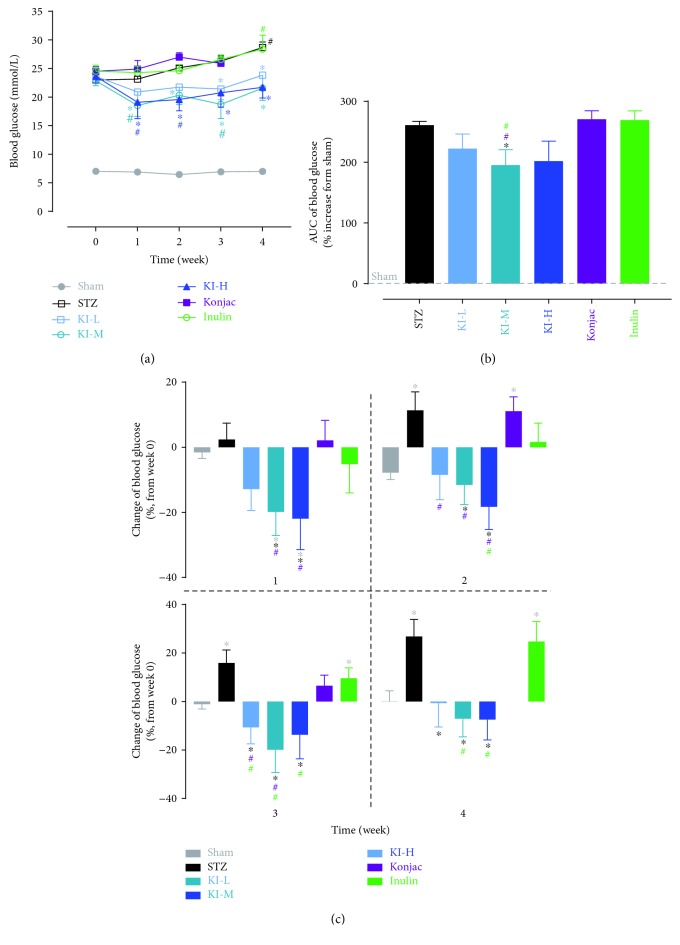
A blood glucose level was monitored over 4 weeks after starting treatments of konjac, inulin, or KI composition at low, medium, and high doses in STZ-induced rats or sham controls (a). 4-week area under curve (b) for each group (increased over a sham group) and changes of the blood glucose level (% increase or decrease from the baseline value at week 0) for each group at each week (c) were also analyzed. *N* = 10 rats, and data were presented as mean ± SEM. ^∗^*P* < 0.05, the konjac-, inulin-, or KI-treated groups were compared with the STZ group using Bonferroni's multiple comparisons test following two-way ANOVA with repeated measures (a). ^#^*P* < 0.05; posttreatment thresholds were compared with pretreatment baselines at week 0 using Dunnett's multiple comparisons test following two-way ANOVA with repeated measures (a). For (b) and (c), ^∗^*P* < 0.05, the konjac-, inulin-, or KI-treated groups were compared with the sham or STZ groups or the STZ group compared with the sham group, and ^#^*P* < 0.05, the KI-treated group was compared with the konjac- or inulin-treated groups using Bonferroni's multiple comparisons test following one-way ANOVA (b) or two-way ANOVA with repeated measures (c). Symbols were represented by the respective group colors that are significantly different from the comparing group.

A blood glucose level was monitored over 4 weeks after starting treatments of konjac, inulin, or KI composition at low, medium, and high doses in STZ-induced rats or their sham controls. All KI compositions showed significant reduction in the blood glucose level than the STZ group during 4-week treatment (from 3 to 4 weeks for KI-L and from 1 to 4 weeks both for KI-M and KI-H); however, no difference was seen between the STZ group and the konjac or inulin group (Figure 1(a)), indicated that konjac and inulin extracts, when combined, were more efficient in decreasing the blood glucose level than a single component alone. The blood glucose level was reduced mostly in the KI-M group, showed by % of AUC increased over sham (Figure 1(b)). When changes of the blood glucose level from baseline were compared at each week, the KI-treated groups had reduction from baseline (<0%) for all 4 weeks, while the konjac, inulin, and STZ groups had increased over baseline (>0%) for all 4 weeks (Figure 1(c)).

### 3.2. Effect of Konjac, Inulin Extracts, and KI Composition on Oral Glucose Tolerance in STZ-Induced Diabetic Rats

**Figure 2:**
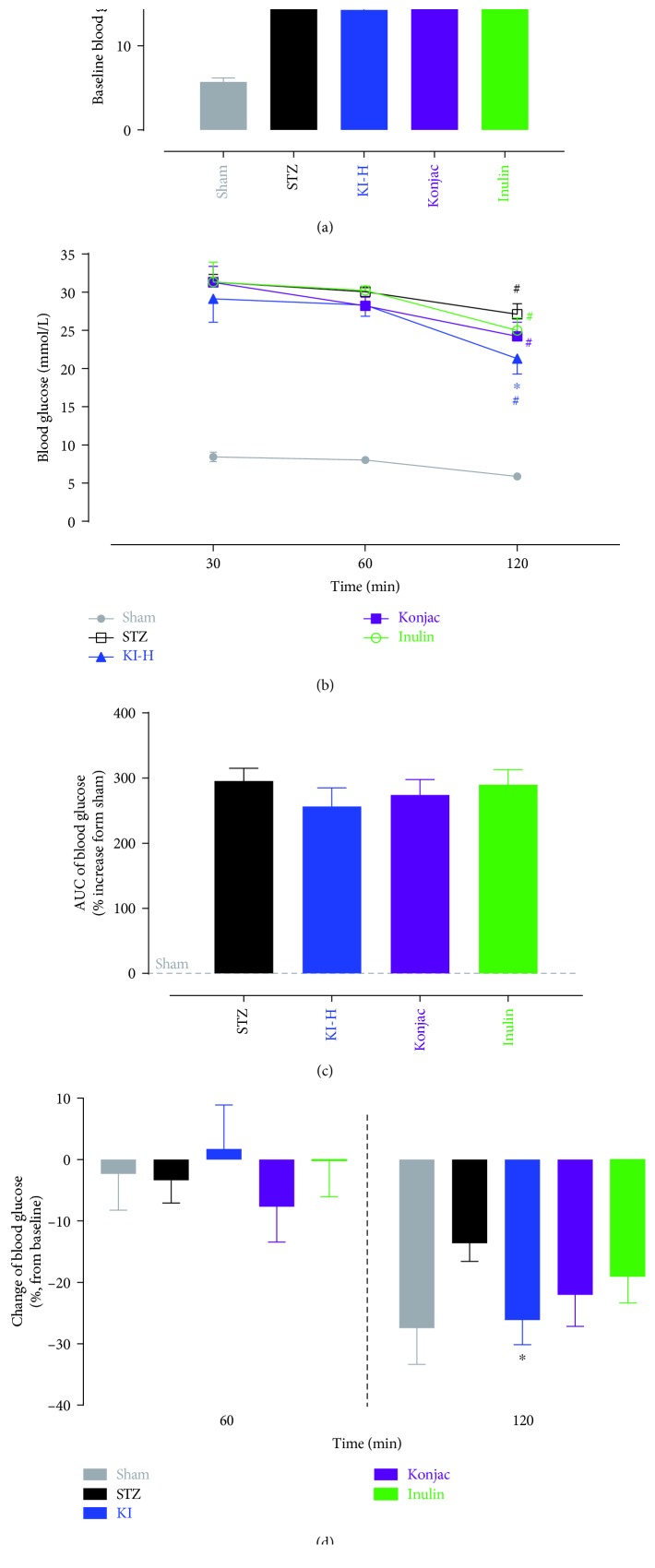
A glucose tolerance study in sham and STZ-induced rats (fasted for 8 hours) after 24-day treatments of vehicle (sham and STZ), konjac, inulin, or KI composition at high dose. Blood glucose levels of each group were monitored right before (a) and at 30, 60, and 120 min (b) after oral administration of 500 mg/kg glucose. Area under curve (c) from 0.5 to 2 hours (increased over the sham group) and changes of the blood glucose level (% increase or decrease from the baseline value at time 30 min) for each group (d) were also analyzed. *N* = 8 rats; data were presented as mean ± SEM. ^∗^*P* < 0.05, the STZ, konjac, inulin, or KI-high-dose-treated group was compared with the sham or STZ group using Bonferroni's multiple comparisons test following one-way ANOVA (a); ^∗^*P* < 0.05, the KI-high-dose-treated group was compared with the STZ group at each time point (120 min was significant) using Bonferroni's multiple comparisons test following two-way ANOVA with repeated measures (b); and ^#^*P* < 0.05, posttreatment thresholds were compared with the values at time 30 min using Dunnett's multiple comparisons test following two-way ANOVA with repeated measures (b). No significant difference in AUC was detected between the groups (c); ^∗^*P* < 0.05, the KI-high-dose group was compared with the STZ group using Bonferroni's multiple comparisons test following two-way ANOVA with repeated measures (d). Symbols were represented by the respective group colors that are significantly different from the comparing group.

A glucose tolerance study was performed in sham and STZ-induced rats (fasted for 8 hours) after 24-day treatments of vehicle (sham and STZ groups), konjac, inulin, or KI composition at high dose (KI-H group). The blood glucose levels were monitored before (baseline) and at 30, 60, and 120 min after oral administration of 500 mg/kg D-glucose (Figure 2). The baseline blood glucose levels of all STZ-induced diabetic rats were significantly higher than the sham rats, and the KI-high-dose-treated group but not the konjac or inulin group had a lower blood glucose level than the STZ group (Figure 2(a)). At 120 min (but not 30 or 60 min) after oral 500 mg/kg D-glucose challenge, the KI-high-dose-treated group (but not the konjac or inulin group) had a significant lower blood glucose level than the STZ group and all groups with STZ-induced rats had a decreased blood glucose level compared with 30 min after oral D-glucose administration (Figure 2(b)). However, no significant difference in AUC (from 30 min to 120 min, increased over sham) was found between the groups. When changes of the blood glucose level from baseline were compared at 120 min, the KI-high-dose-treated group (but not the konjac or inulin group) had significant higher reduction from baseline than the STZ group (Figure 2(c)).

### 3.3. Effect of Konjac, Inulin Extracts, and KI Composition on Biochemical Parameters and Insulin Expression in STZ-Induced Diabetic Rats

**Figure 3:**
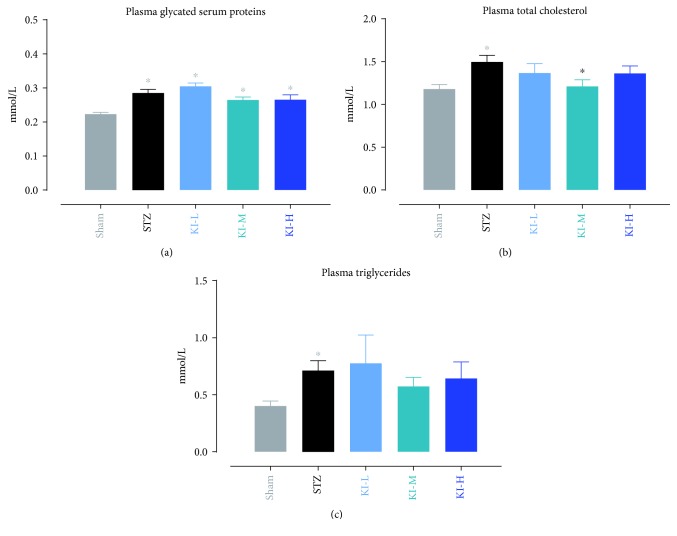
Levels of plasma glycated serum proteins (a), plasma total cholesterol (b), and plasma triglycerides (c) were measured in STZ-induced rats treated with konjac, inulin, or KI composition at low, medium, and high doses or in sham controls, after daily treatment for 28 days. *N* = 9 to 10 rats; data were presented as mean ± SEM. ^∗^*P* < 0.05, the konjac-, inulin-, or KI-treated groups were compared with the STZ and sham groups or the STZ group compared with the sham group using Bonferroni's multiple comparisons test following one-way ANOVA (a–c). Symbols were represented by the respective group colors that are significantly different from the comparing group.

**Figure 4:**
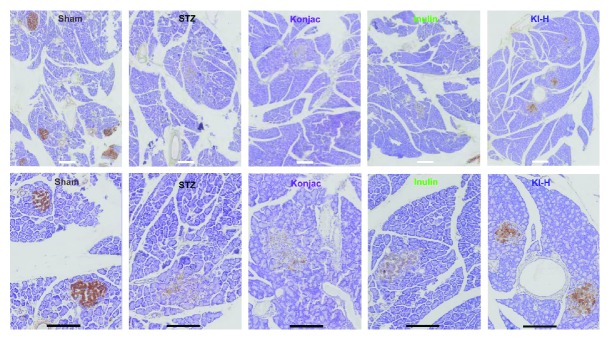
Illustration of hematoxylin and eosin (HE) staining of pancreatic islet slices in vehicle-treated sham SD rats or vehicle, konjac, inulin, and KI-high-dose-treated STZ-induced rats, after daily treatment for 28 days, in original (first row) or enlarged scale (second row). White bars in the first row represent 100 *μ*m, while black bars in the second row represent 50 *μ*m. Insulin was stained in dark brown color. There was a notable weakened insulin staining trend in the STZ model group compared with the sham group, and such weakened insulin staining was partially reversed in the KI-high-dose-treated group.

After daily treatment for 28 days, the levels of plasma glycated serum proteins (GSP), plasma total cholesterol, and plasma triglycerides were measured in STZ-induced rats treated with konjac, inulin, or KI composition at low, medium, and high doses or in sham controls. KI-L, KI-M, KI-H, and STZ groups had a significant higher plasma GSP level than the sham groups; however, STZ-treated groups are not different from each other (Figure 3(a)). The plasma total cholesterol level in the STZ group was significantly higher than the sham and KI-M groups (Figure 3(b)). Only the STZ group had a significant higher plasma triglyceride level than the sham group (Figure 3(c)), suggested that konjac, inulin, or KI composition may be effective in reducing the blood triglyceride level in STZ-induced diabetic rats. After daily treatment for 28 days of each diet, insulin expressions were illustrated using immunohistochemistry in hematoxylin and eosin staining of pancreatic islet slices (Figure 4). Compared with sham controls, insulin expressions were markedly reduced in STZ-induced rats treated with vehicle, konjac, or inulin extracts; nevertheless, such reduction was found to be partially reversed in KI-high-dose-treated STZ-induced rats indicated the improvement in insulin production function of islet cells (Figure 4).

### 3.4. Effect of Konjac, Inulin Extracts, and KI Composition on a Blood Glucose Level of db/db Mice

**Figure 5:**
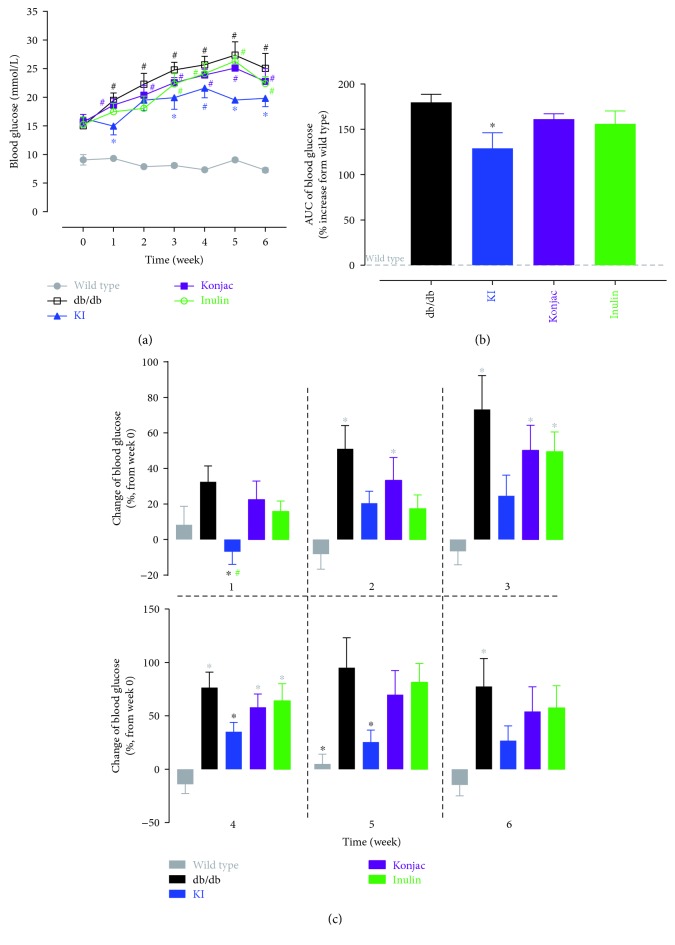
A blood glucose level was monitored over 6 weeks after starting treatments of konjac, inulin, or KI composition in db/db mice or wild-type controls (a). 6-week area under curve (b) for each group (increased over the wild-type group) and changes of the blood glucose level (% increase or decrease from the baseline value at week 0) for each group at each week (c) were also analyzed. *N* = 7 mice; data were presented as mean ± SEM. ^∗^*P* < 0.05, the konjac-, inulin-, or KI-treated groups were compared with the db/db group using Bonferroni's multiple comparisons test following two-way ANOVA with repeated measures (a). ^#^*P* < 0.05, posttreatment thresholds were compared with pretreatment baselines at week 0 using Dunnett's multiple comparisons test following two-way ANOVA with repeated measures (a). For (b) and (c), ^∗^*P* < 0.05, the konjac-, inulin-, or KI-treated groups were compared with the wild-type or db/db groups, and ^#^*P* < 0.05, the KI-treated group was compared with the inulin-treated group using Bonferroni's multiple comparisons test following one-way ANOVA (b) or two-way ANOVA with repeated measures (c). Symbols were represented by the respective group colors that are significantly different from the comparing group.

To evaluate the effect of konjac, inulin extracts, and KI composition on type 2 diabetes, db/db mice were used. A blood glucose level was monitored over 6 weeks after starting treatments of each diet (Figure 5). The KI composition showed a significant reduction in the blood glucose level than the STZ group at weeks 1, 3, 5, and 6 (Figure 5(a)). Blood glucose increase (from baseline) was seen in most time points from 1 to 6 weeks for STZ, konjac, and inulin groups but not for the KI group (Figure 5(a)). The blood glucose level was reduced mostly in the KI group, which was significantly lower than the STZ group, showed by % of AUC increased over sham (Figure 5(b)). When changes of the blood glucose level from baseline were compared at each week, the KI group had reduction from baseline (<0%) at week 1 and a significant difference was also seen between the KI group and the STZ or inulin group (Figure 5(c)). Also, the KI group had significantly less increase of the blood glucose level than the STZ group at weeks 4 and 5 (Figure 5(c)).

### 3.5. Effect of Konjac, Inulin Extracts, and KI Composition on the Body Weight of db/db Mice

**Figure 6:**
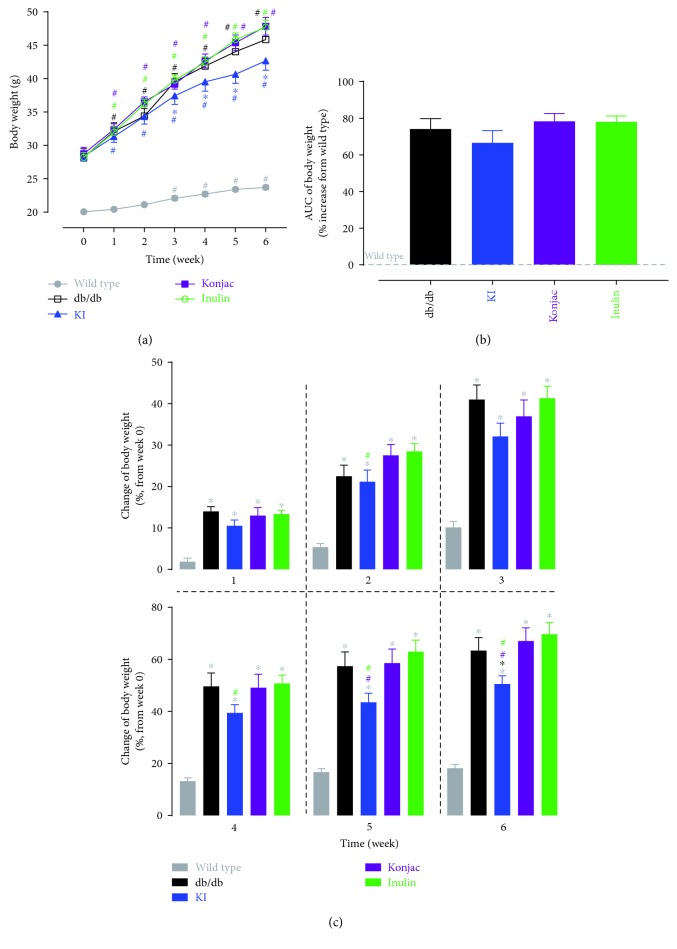
Body weight was monitored over 6 weeks after starting treatments of konjac, inulin, or KI composition in db/db mice or wild-type controls (a). 6-week area under curve (b) for each group (increased over the wild-type group) and changes of the body weight (% increase or decrease from the baseline value at week 0) for each group at each week (c) were also analyzed. *N* = 7 mice; data were presented as mean ± SEM. ^∗^*P* < 0.05, the konjac-, inulin- or KI-treated groups were compared with the db/db group using Bonferroni's multiple comparisons test following two-way ANOVA with repeated measures (a). ^#^*P* < 0.05, posttreatment thresholds were compared with pretreatment baselines at week 0 using Dunnett's multiple comparisons test following two-way ANOVA with repeated measures (a). For (b) and (c), ^∗^*P* < 0.05, the konjac-, inulin-, or KI-treated groups were compared with the wild-type or db/db groups, and ^#^*P* < 0.05, the KI-treated group was compared with the konjac- or inulin-treated groups using Bonferroni's multiple comparisons test following one-way ANOVA (b) or two-way ANOVA with repeated measures (c). Symbols were represented by the respective group colors that are significantly different from the comparing group.

The KI composition showed a significant reduction in the body weight than the STZ group from weeks 3 to 6 (Figure 6(a)). The body weight was reduced mostly in the KI group however was not significantly lower than the STZ group (although the trend was seen), showed by % of AUC increased over sham (Figure 6(b)). When changes of the body weight from baseline were compared at each week, the KI group showed significantly less increase of the body weight than the konjac and/or inulin group at weeks 2, 4, 5, and 6 (Figure 6(c)).

### 3.6. Effect of Konjac, Inulin Extracts, and KI Composition on Blood Triglycerides of db/db Mice

**Figure 7:**
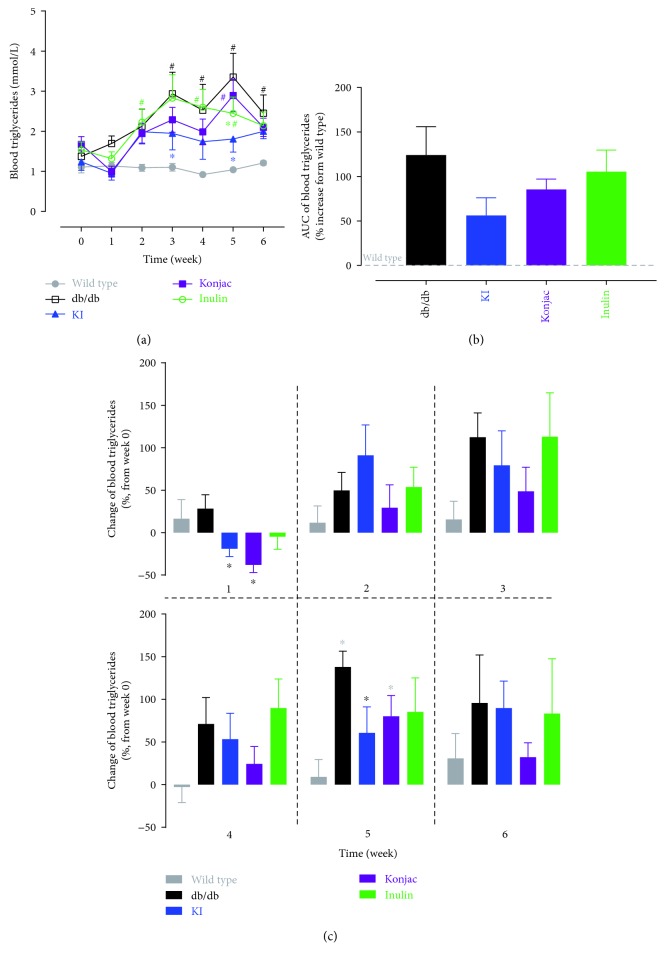
Blood triglyceride levels were monitored over 6 weeks after starting treatments of konjac, inulin, or KI composition in db/db mice or wild-type controls (a). 6-week area under curve (b) for each group (increased over the wild-type group) and changes of blood triglycerides (% increase or decrease from the baseline value at week 0) for each group at each week (c) were also analyzed. *N* = 7 mice; data were presented as mean ± SEM. ^∗^*P* < 0.05, the konjac-, inulin- or KI-treated groups were compared with the db/db group using Bonferroni's multiple comparisons test following two-way ANOVA with repeated measures (a). ^#^*P* < 0.05, posttreatment thresholds were compared with pretreatment baselines at week 0 using Dunnett's multiple comparisons test following two-way ANOVA with repeated measures (a). For (b) and (c), ^∗^*P* < 0.05, the inulin- or KI-treated groups were compared with the wild-type or db/db groups using Bonferroni's multiple comparisons test following one-way ANOVA; no significant difference was found when comparing the KI-treated group with the konjac- or inulin-treated groups. Symbols were represented by the respective group colors that are significantly different from the comparing group.

The KI composition showed a significant reduction in blood triglycerides than the STZ group at weeks 3 and 5 (Figure 7(a)). A blood triglyceride level was reduced mostly in the KI group however was not significantly lower than the STZ group (although the trend was seen), showed by % of AUC increased over sham (Figure 7(b)). When changes of blood triglycerides from baseline were compared at each week, the KI and konjac groups had reduction from baseline (<0%) at week 1 and significant difference was also seen between these two groups and the STZ group (Figure 7(c)). Also, the KI group showed significantly less increase of the blood triglyceride level than the STZ group at week 5 (Figure 7(c)). Correlated the efficacy of KI compositions in STZ-induced diabetic rats, KI composition showed advantage over konjac or inulin extract alone, in both type 1 and type 2 diabetes.

## 4. Discussion

The modern diet is much lower in nondigestible carbohydrates (dietary fibers) than all previous diets in human history, potentially contributing to changes in the gut microbiota, which subsequently leading to increases in chronic lifestyle diseases [9]. In order to reverse such undesirable changes and to improve metabolic function, modulation of the gut microbiota composition has received tremendous attention. It was found that the modulation of the gut microbiota (characterized mainly by an increased abundance of the *genus Akkermansia*) might contribute to the antidiabetic effect of metformin, one of the most widely prescribed therapeutic agents for type 2 diabetes [10]. One of the latest strategies is prebiotics, which typically refer to “selectively fermented nondigestible food ingredients or substances that specifically support the growth and/or activity of health-promoting bacteria that colonize the gastrointestinal tract” [11]. Prebiotics are known to be beneficial to conditions of metabolic diseases and overweight [12–15].

Inulin was considered as one of the earliest accepted and most studied prebiotics [11]. For instance, the administration of inulin has a broad effect on the gut microbial ecosystem and regulated the abundance of microorganisms within microbiota in genetically obese mice [16]. The konjac extracts, konjac glucomannan, and konjac oligosaccharides were also thought to play a role as prebiotics by inducing beneficial physiological effects [3]. It was suggested that prebiotics such as inulin and konjac glucomannan and oligosaccharides had highly selective effects on the human gut microbiota, increasing mainly the population levels of *bifidobacteria* and *lactobacilli* while decreasing the cell numbers of the *genus Bacteroides*, *Clostridia*, and *Fusobacteria* [17], while most dietary fibers do not induce selective changes in the gut microbiota [18] and thus not considered as prebiotics.

In the current study, by including two ingredients of prebiotics, which pass undigested through the upper part of the gastrointestinal tract and stimulate the growth of health-promoting bacteria that colonize the large bowel, we were able to show that the KI composition is effective in reducing hyperglycemia and blood triglycerides in both type 1 and 2 diabetes and preventing obesity in db/db mice, implying KI composition's potential indication in metabolic and cardiovascular diseases, besides its primary role as a food supplement to ameliorate the insufficiency in dietary fibers.

Numerous studies have shown that reduction in endogenous antioxidative substances combined with the increase in free radical production was associated with oxidative stress and damage of cell components in diabetes, and it is hypothesized that an antioxidant therapy could help to prevent diabetic complications, such as by suppressing the peroxidation chain reaction [19]. As from a safe organic source, the konjac extract, konjac oligosaccharide, was found to bare a property of a free radical scavenger that suppresses the peroxidation and attenuates upregulated the environmental nitric oxide level in islets of STZ-induced diabetic rats, while at the same time improved function of insulin secretion [4]. Similarly, with low calorie, inulin lowers the blood lipid and blood glucose level [5, 17], as well as exerts a non-insulin-dependent therapeutic effect on diabetes through increasing the total antioxidant capacity and superoxide dismutase activities [20]. Hence, synergy between konjac and inulin extracts might be achieved in KI composition based on their common antioxidative abilities with complementary mechanisms [4, 20]. Such rationale to generate effective combinations has been successfully demonstrated in our previous attempt in the treatment of neurological disorders [21–23]. However, future studies are still required to illustrate whether the protective effect of konjac plus inulin is mediated through enhanced insulin regeneration in pancreatic islets.

In conclusion, we showed that daily use of KI composition is efficient in decreasing blood sugar and blood fat, improving the insulin production in islets, or decreasing obesity in STZ-induced diabetic rats or in db/db mice. Such effects were better than a single ingredient of konjac or inulin extract. This study provided experimental basis for further development of KI composition as a food supplement for diabetic or diabetic high-risk population.

## Data Availability

The data of this study are available from the corresponding authors on reasonable request.
